# From Sound to Movement: Mapping the Neural Mechanisms of Auditory–Motor Entrainment and Synchronization

**DOI:** 10.3390/brainsci14111063

**Published:** 2024-10-25

**Authors:** Marija Pranjić, Thenille Braun Janzen, Nikolina Vukšić, Michael Thaut

**Affiliations:** 1 Music and Health Science Research Collaboratory, University of Toronto, Toronto, ON M5S 1C5, Canada; 2 Division of Developmental Medicine, Boston Children’s Hospital, Harvard Medical School, Boston, MA 02115, USA; 3 Center of Mathematics, Computing and Cognition, Universidade Federal do ABC, São Bernardo do Campo, Sao Paulo 09606-045, Brazil; 4 Independent Researcher, Zagreb 10000, Croatia; 5 Faculty of Medicine, Institute of Medical Science and Rehabilitation Research Institute, University of Toronto, Toronto, ON M5S 1A8, Canada

**Keywords:** auditory–motor entrainment, sensorimotor synchronization, neural entrainment, auditory perception, music, rhythm, finger tapping, scoping review

## Abstract

Background: Humans exhibit a remarkable ability to synchronize their actions with external auditory stimuli through a process called auditory–motor or rhythmic entrainment. Positive effects of rhythmic entrainment have been demonstrated in adults with neurological movement disorders, yet the neural substrates supporting the transformation of auditory input into timed rhythmic motor outputs are not fully understood. We aimed to systematically map and synthesize the research on the neural correlates of auditory–motor entrainment and synchronization. Methods: Following the PRISMA-ScR guidelines for scoping reviews, a systematic search was conducted across four databases (MEDLINE, Embase, PsycInfo, and Scopus) for articles published between 2013 and 2023. Results: From an initial return of 1430 records, 22 studies met the inclusion criteria and were synthesized based on the neuroimaging modality. There is converging evidence that auditory–motor synchronization engages bilateral cortical and subcortical networks, including the supplementary motor area, premotor cortex, ventrolateral prefrontal cortex, basal ganglia, and cerebellum. Specifically, the supplementary motor area and the basal ganglia are essential for beat-based timing and internally guided rhythmic movements, while the cerebellum plays an important role in tracking and processing complex rhythmic patterns and synchronizing to the external beat. Self-paced tapping is associated with additional activations in the prefrontal cortex and the basal ganglia, suggesting that tapping in the absence of auditory cues requires more neural resources. Lastly, existing studies indicate that movement rate and the type of music further modulate the EEG power in the alpha and beta frequency bands. Conclusions: These findings are discussed in the context of clinical implications and rhythm-based therapies.

## 1. Introduction

Whether we are synchronizing our steps to the beat of music or adjusting our speech patterns during conversational turn-taking, auditory–motor entrainment plays a crucial role in generating predictive signals that allow for the precise alignment of movements with anticipated temporal structures [1,2,3]. Entrainment refers to the coupling of two or more entities whereby an independent oscillator is influenced by another oscillator, resulting in synchronization in frequency, phase, or both [4,5,6]. This phenomenon is evident in both physical (e.g., pendulum clocks) and biological systems (e.g., fireflies, circadian rhythms), whereby the motion or signal frequency of one system synchronizes with that of another [7,8]. In the context of human motor behavior, entrainment involves the temporal coordination between body movements and periodic stimuli, including auditory, visual, tactile, multimodal, or social signals [2,9].

Therefore, auditory–motor synchronization (AMS) can be defined as the production of rhythmic motor actions synchronized to the periodic structure of an acoustic stimulus whereby the rhythmic properties of the external cues entrain the internal oscillations of the brain and the body [4,10]. In particular, the rhythmic structure of music, characterized by its regular temporal patterns and hierarchical organization, inherently encourages coupling between perception and action [10]. The seamless integration of movement with music, whether it is tapping one’s foot to a catchy tune or swaying to the rhythm of a song, highlights the intrinsic connection between auditory perception and motor control [11].

The ability to extract temporal regularities and align motor responses to the periodic structure of rhythmic sequences, such as the pulse of the music, is considered a near-universal trait in humans [12] and is only rarely observed in non-human species [13,14,15,16]. Existing studies suggest that the maturation of the motor system and the establishment of functional connections between auditory and motor areas are essential for the development of AMS [17,18]. Notably, research has shown that this skill can be improved with musical training. For example, musicians exhibit greater accuracy in tasks involving synchronization to the beat of music than individuals without musical training [3,19,20,21,22,23,24].

The pervasive tendency to move in response to music finds its roots in the structure and functioning of the human brain [25]. Modern neuroimaging techniques have revealed a significant interaction between the auditory and motor systems during movement synchronization to predictable rhythmic stimuli [7]. Moreover, the mere perception of temporally structured stimuli, even in the absence of overt movements, triggers activity in regions associated with motor control, such as the basal ganglia and cortical motor areas [26,27,28,29]. Over the past decades, the number of publications regarding the neural mechanisms underlying auditory–motor synchronization has steadily increased. These studies have predominantly employed finger-tapping paradigms, which offer a well-controlled way to capture the transformation of auditory input into a timed rhythmic motor output.

### 1.1. The Finger-Tapping Paradigms

The finger-tapping paradigm typically requires participants to synchronize the movement of the index finger of their dominant hand to computer-generated isochronous pacing sequences, such as metronome clicks, single tones, or music, hence examining motor responses entrained to the onset of sensory stimuli [30]. Drum strokes [31] or foot/hand tapping may also be implemented [30]. While finger-tapping is considered a simple task for most neurotypical individuals, synchronizing one’s actions with a rhythmic stimulus has been shown to engage complex timing processes and sensorimotor integration mechanisms. Specifically, AMS involves extracting and maintaining the rhythmic period of stimuli, executing movements with the support of reliable internal timekeeping systems, and engaging both anticipatory and adaptation processes [31,32].

Furthermore, AMS generally operates on a sub-second timescale, particularly when participants tap at a low level of the metric hierarchy [33]. Research has shown that synchronized tapping to a beat is accurately maintained at intervals ranging from 200 to 1800 ms, with optimal being accuracy observed around 600 ms [30], while the variability of self-selected rates may be influenced by both intrinsic and extrinsic factors (for a review, see [34]). Furthermore, a stable synchronization state is usually achieved within two to three stimulus repetitions and can be maintained reliably over extended timing sequences [32].

Researchers may employ different finger tapping tasks to assess AMS abilities [35]. Paced tapping generally involves synchronizing finger taps with a regular, evenly spaced sequence of auditory stimuli, typically presented by a metronome or a musical excerpt. Therefore, paced tapping tasks heavily rely on rhythmic entrainment processes. Paced tasks are often contrasted with unpaced tapping, wherein participants produce finger taps at a self-selected pace (i.e., their spontaneous rate), providing insights into their intrinsic timing abilities for generating and maintaining rhythm. Internal timing can also be studied using a synchronization–continuation task [36], which requires participants to initially synchronize finger taps with an external metronome. Then, after a few presentations, the external pacing stops and participants are instructed to maintain their movement rate until the end of the trial. Thus, the continuation phase examines processes responsible for maintaining the representation of an external rhythmic stimulus that is no longer available.

The distinction between tapping tasks is important for understanding the cognitive and neural processes underlying AMS [3]. In behavioral studies, synchronization performance is typically assessed by computing the synchronization error (asynchrony) and absolute period error, while the average of the inter-tap intervals (ITIs) and their variability (e.g., the mean coefficient of variation) are typically reported for unpaced movements. Asynchrony refers to the time difference between the tap and the onset of the external rhythm, whereas the degree of variability of the asynchrony (measured by the standard deviation of asynchronies) is an index of tapping stability [3]. Research has shown that motor responses often occur slightly before the expected stimulus onset, a phenomenon known as negative mean asynchrony [30,37]. Despite variations in the size of asynchronies across different experimental conditions and notable inter-individual differences, the consistent pattern of taps preceding the external pacing signal indicates that predictive anticipatory mechanisms are at play in AMS [31]. Moreover, sensorimotor adaptation is crucial for maintaining precise yet flexible movements. Error correction processes, known as phase correction and period correction, compensate for timing errors [31,38]. These distinct correction processes are often studied by introducing tempo perturbations, which reveal the intricate mechanisms underlying AMS adaptations (for a review, see [3,30,31]). Taken together, studies employing finger-tapping paradigms present a well-controlled way to gain insight into the neural correlates of auditory–motor entrainment and synchronization.

### 1.2. Aims

The aim of this review was to systematically map and synthesize the existing neurophysiological and neuroimaging research on auditory–motor entrainment and synchronization (AMS) in neurotypical adults. Specifically, we focused on finger-tapping paradigms to enhance clarity and comparability between the included studies as well as to supplement and extend earlier reviews on AMS that were focused on finger-tapping paradigms [3,30]. We addressed the following questions: (1) What is known from the literature about the neural mechanisms of auditory–motor entrainment and synchronization in neurotypical adults? (2) What neurobiological substrates characterize auditory-paced movements compared to unpaced movements?

## 2. Materials and Methods

The scoping review was conducted using the Preferred Reporting Items for Systematic Reviews and Meta-Analyses Extension for Scoping Reviews (PRISMA-ScR) criteria: Checklist and Explanation guidelines [39], in compliance with the Joanna Briggs Institute’s methodological framework [40].

### 2.1. Eligibility Criteria

Articles were eligible if they (1) included neurotypical, healthy adults (>18 years); (2) involved an isochronous auditory–motor synchronization task (without perturbations); (3) reported neurophysiological or neuroimaging outcomes; (4) were published after 2012; (5) were published in English; (6) were categorized as original research. Studies involving animals or clinical populations were excluded, as well as review articles, conference proceedings, and book chapters. The publication date was selected to supplement and extend the last comprehensive review on AMS using finger-tapping paradigms [3] and thus capture the emerging evidence in the field.

### 2.2. Search Strategy

The search was carried out across four major databases, including MEDLINE, Embase, PsycInfo, and Scopus, for articles published between January 2013 and January 2023. The PCC (Population, Concept, Context) mnemonic was used to formulate the search strategy. The search strategy was developed by the first author with assistance from a research librarian. Terms such as “neurotypical” or “healthy adults” were used to define the population, the terms “auditory–motor entrainment” or “auditory–motor synchronization” were used to describe the concept, and the search terms “finger-tapping” or “hand-tapping” were used to specify the context. Subject headings were adapted for each database. Table 1 provides an example of the relevant search terms implemented in MEDLINE (see Supplementary Table S1 for the full electronic search strategy). Additionally, we examined the reference lists of selected studies and performed a backward and forward citation search using Google Scholar to identify publications that were not initially included in the database search.

### 2.3. Screening and Data Extraction

The screening and data extraction were performed by two independent reviewers (M.P. and N.V.). The appropriate filtering of titles, abstracts, and full-text publications was completed using the Covidence software. Relevant studies were evaluated, and discrepancies between the reviewers were resolved by consensus. Full-text exclusion criteria were further applied, encompassing studies that involved listening tasks only [41], self-initiated sounds [42], rhythm reproduction tasks [43], irregular/unpredictable rhythmic sequences only [44], and those where neuroimaging was not recorded during the AMS task [45]. We systematically extracted the following information from the included papers: publication details, sample characteristics (i.e., age, sex, sample size), task characteristics (i.e., paced vs. unpaced finger tapping), brain imaging methods (e.g., EEG, MRI, etc.), and key findings. Data from the selected studies were collated, summarized, and reported based on the brain imaging modality, highlighting both the temporal and spatial characteristics.

## 3. Results

**Figure 1:**
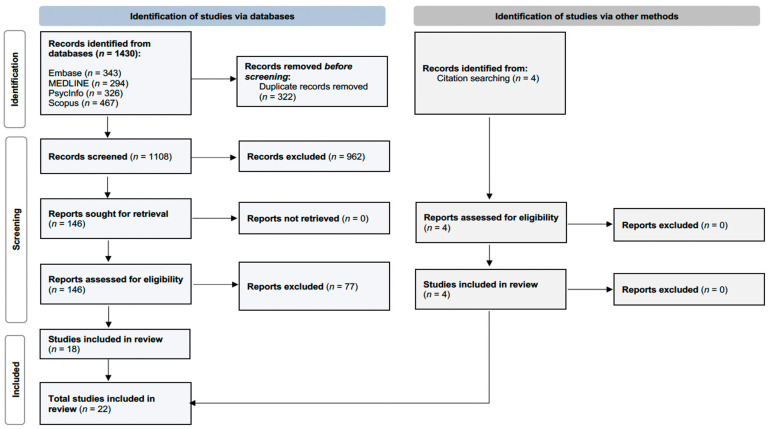
PRISMA Flow Diagram of the article selection process.

A total of 1430 records were initially identified from searches across four electronic databases (294 records in MEDLINE, 343 in Embase, 326 in PsycInfo, and 467 in Scopus). After the removal of duplicate entries, 1108 studies were screened based on the title and abstract, of which 962 did not meet the inclusion criteria. A full review was conducted for the remaining 146 records, and 18 papers met the inclusion criteria. Four additional papers were identified through citation searching; therefore, a total of 22 papers were included in this review (see Figure 1). About half of the included studies employed electroencephalography (EEG) (*n* = 10), followed by magnetic resonance imaging (MRI) (*n* = 5) and functional near-infrared spectroscopy (fNIRS) (*n* = 2). We also identified one study using positron emission tomography (PET), transcranial magnetic stimulation (TMS), and transcranial direct current stimulation (tDCS), as well as two studies that employed a combination of multiple neuroimaging techniques.

The following sections synthesize key findings from the selected articles based on the neurophysiological and neuroimaging methodology implemented (see Table 2). First, we discuss EEG findings due to their temporal sensitivity to both external and internal stimuli. Then, we report on measurements with high spatial resolution, including MRI, fNIRS, and PET. Lastly, we synthesize studies that utilize perturbation approaches (i.e., TMS and tDCS) and those that combine neuroimaging techniques.

### 3.1. Electroencephalography (EEG) Studies

Brain oscillations detected in EEG result from fluctuations in synchronized neuronal activity within the cortex [46]. Analyzing oscillatory brain dynamics reveals crucial information about the time-sensitive excitatory and inhibitory mechanisms that underlie various cognitive and sensory processes [47]. Brain oscillations are typically categorized into distinct frequency bands such as delta (0–4 Hz), theta (4–7.5 Hz), alpha (8–13 Hz), beta (13–30 Hz), and gamma (>30 Hz). When these neural oscillatory signals are segmented and synchronized with recurring events, such as auditory tones, the averaged data across trials reveal phase-locked evoked responses [48,49]. Therefore, the study of neural oscillations plays a key role in understanding the neurophysiological markers of auditory–motor entrainment and synchronization. Among the studies included, ten employed EEG paradigms to examine the brain signatures involved in temporal processing.

To explore the oscillatory brain dynamics during AMS, two studies investigated steady-state-evoked potentials (SS-EPs) during sensorimotor synchronization [50,51]. SS-EPs are frequency domain representations of stable neural responses to periodic auditory stimuli, thus serving as a powerful tool for studying continuous brain activity modulations in sustained rhythmic tasks without isolating specific events [52].

In the study by Nozaradan et al. [50], participants listened to a 2.4 Hz auditory beat and tapped their hand on every second beat. The EEG data revealed three key findings: first, a 2.4 Hz SS-EP, reflecting beat-related entrainment; second, a 1.2 Hz SS-EP, corresponding to movement-related entrainment; third, the emergence of a 3.6 Hz SS-EP during tapping, which is compatible with a nonlinear interaction between sensory and motor processes. Additionally, phase coupling between beat- and movement-related activities was observed, along with a selective enhancement of beat-related activity in the hemisphere contralateral to the tapping hand, suggesting a top-down influence of motor-related activity on auditory beat processing. Building on these findings, another SS-EP study by De Pretto et al. [51] compared auditory-paced, self-paced, and passive listening conditions and found higher activations of the left inferior frontal gyrus (IFG) during synchronization as well as greater bilateral inferior parietal lobule (IPL) activation during self-paced finger tapping. These findings highlight the important role of the left IFG in perception–action coupling.

In a study measuring cortical and muscular activity, Nijhuis et al. [53] compared real and imagined movement synchronization to visual and auditory cues to examine the processes of movement preparation and execution during AMS. The study found that cortico-muscular coupling occurred in the beta band frequencies during synchronization with both visual and auditory stimuli as well as during imagined synchronization with visual stimuli. This finding suggests that cortico-muscular coupling reflects changes in both movement execution and preparation. Indeed, changes in beta frequency (13–30 Hz) are closely associated with sensorimotor behavior and play a key role during tasks requiring precise timing and rhythmic coordination. Furthermore, beta oscillations subserve auditory–motor entrainment by facilitating real-time adjustments and connections between auditory cues and motor output. Notably, Nijhuis et al. [53] found that the amplitudes during imagined movements were lower than those during real (i.e., active) synchronization. This suggests that, while imagined movements engage similar neural mechanisms, the level of neural activation is reduced, reflecting both the different temporal dynamics and the potentially lower attentional demands during imagined synchronization compared to real synchronization.

Several studies have also delved into the link between neural oscillations and synchronization accuracy, further elucidating the mechanisms at play. Rosso et al. [54] expanded on the SS-EP approach by measuring the stability of neural entrainment through dynamic phase adjustments during movement tasks. Their results showed that greater stability in the neural component corresponded to more stable and accurate synchronization. Furthermore, Bavassi et al. [55] identified the neurophysiological markers of sensorimotor synchronization, showing that similar neural mechanisms may underpin both the correction of large asynchronies in periodic tasks and the resynchronization of tapping after perturbations. Their findings suggest that an EEG component related to the perception of asynchrony can predict future synchronization accuracy and that synchronization error processing occurs within a consistent time window following each stimulus.

Perceptual and motor components inherent in AMS were further studied using an oddball paradigm [56], which revealed that a larger N1-P2-evoked potential complex occurred in response to the unexpected (i.e., deviant) beats compared to temporally predictable ones. Moreover, a stronger response to deviants was associated with better AMS performance. This finding highlights the role of adaptive neural mechanisms, particularly the N1-P2 complex, in responding to irregular rhythmic stimuli and their critical link to synchronization accuracy. Similar findings were reported by Mathias et al. [57], who investigated the influence of rhythm complexity on AMS. They reported that the EEG spectral density was more enhanced during AMS compared to listening-only conditions, wherein prominent peaks occurred at or near target frequencies of 0.94, 1.89, and 2.84 Hz. Their results also indicated that neural measures of entrainment decreased as rhythmic complexity increased during both listening and synchronizing tasks, particularly in auditory and motor regions of interest, suggesting that rhythmic complexity directly modulates both behavioral and neural measures.

Studies by Stegemöller and colleagues [58,59] further explored the influence of task complexity, such as tapping rate and music style, on cortical motor activity. In their earlier study, Stegemöller et al. [58] observed increased beta-band power during slow tapping (70 BPM) and alpha-band power during fast tapping (140 BPM) to music compared to tone-only, suggesting that moving to music engages larger neuronal networks. Interestingly, they also found that musicians exhibited increased beta-band power across all conditions compared to individuals without musical training, indicating that musical training may enhance motor cortical activity, particularly in the beta band, which could contribute to improved rhythmic processing and motor performance. In a subsequent study, Stegemöller et al. [59] showed that the type of music further modulated EEG power in addition to movement rate. Specifically, they reported significantly decreased alpha and beta power during “activating music” compared to “relaxing music”. These results underscore the importance of considering both the style of music and the tempo when designing therapeutic applications, as these factors can differentially impact motor cortical activity and potentially optimize motor performance outcomes.

Lastly, a study by Crasta et al. [49] demonstrated that listening to auditory rhythms before producing rhythmic movements can enhance the entrainment process and improve motor control. This enhancement was evidenced by significantly lower evoked power in sensorimotor and predictive timing regions in participants who received auditory priming before the AMS task, particularly in the beta and gamma frequency ranges. The findings suggest that brief auditory training facilitates more efficient neural processing during entrainment, with important implications for interventions using rhythmic auditory stimulation. These results suggest that brief auditory training facilitates more efficient neural processing during entrainment, with important implications for interventions using rhythmic auditory stimulation.

In summary, the IFG and the dorsal stream have been shown to play a crucial role in supporting sound-to-action processes during auditory–motor synchronization. These studies advance our understanding of the mechanisms underlying beat- and movement-related entrainment and demonstrate how task complexity, rhythm, and musical training influence neural oscillations across different frequency bands, particularly in the beta and gamma ranges.

### 3.2. Magnetic Resonance Imaging (MRI), Functional Near-Infrared Spectroscopy (fNIRS), and Positron Emission Tomography (PET) Studies

Neuroimaging techniques with high spatial resolution have been widely employed to examine the roles of cortical and subcortical brain regions in sensorimotor synchronization. The studies reviewed in this section utilize various imaging modalities, including magnetic resonance imaging (MRI), positron emission tomography (PET), and functional near-infrared spectroscopy (fNIRS), each providing unique insights into the neural mechanisms underlying this process. For a comprehensive overview of MRI studies in this domain, see these reviews [60,61,62,63].

Activity within the ventrolateral prefrontal cortex (VLPFC), including the IFG, has been associated with tasks that actively require retrieval and monitoring of the selected beat [64,65,66]. For instance, Witt and Stevens [66] investigated top-down control during different phases of an unimanual, auditory-paced finger tapping task. They compared brain activity during passive listening, synchronization, and continuation tapping, observing increased activity in the right VLPFC during continuation tapping compared to other conditions. Furthermore, participants who performed better at maintaining the tapping pace in the absence of the auditory cue relied more the on top-down control exerted by the prefrontal cortex over motor and sensory regions, whereas those with less accurate performance relied more on sensory-driven, bottom-up control. Moreover, distinct lateralized roles were observed in the prefrontal cortex, contributing to optimal performance, although no significant maturational effects were detected. These findings concur with the earlier hypotheses of top-down control, suggesting that activity in the prefrontal–parietal–temporal working memory network is required for maintaining internal timing during tapping when auditory cues are absent [67]. Miyata et al. [68] showed that individual variations in temporal prediction ability, measured by the prediction/tracking ratio, were positively correlated with activity in the bilateral dorsal premotor cortex (dPMC). Participants with a higher prediction/tracking ratio demonstrated more accurate and precise tapping to metronome beats, suggesting that the dPMC plays a critical role in generating and maintaining accurate temporal predictions during sensorimotor synchronization.

The microstructural properties underlying AMS were studied using diffusion magnetic resonance imaging (dMRI) in a task involving changes in the metric structure [69]. The study showed that enhanced AMS abilities were positively correlated with fractional anisotropy (a measure of microstructural integrity) in the left arcuate fasciculus and temporal callosal fibers. These results indicate that auditory–motor synchronization is mediated by activity in the left frontotemporal and temporal-callosal fibers, which help predict and compare incoming stimuli with motor output. Kung et al. [64] examined brain activity while participants actively tried to find the beat or tapped in synchrony to rhythms that varied in metrical complexity. Findings showed that both tasks recruited a network involving the superior temporal gyrus (STG), premotor cortex, basal ganglia, and VLPFC. Activity in the STG and VLPFC was correlated with both perception and performance, and activation was more strongly modulated by beat strength, with greater activity observed for more metrically complex rhythms with weaker beats. The basal ganglia (BG) were engaged during both tasks but did not show increased activity related to beat strength. Instead, the BG interacted with the VLPFC, particularly during tapping to weaker beats, suggesting that the BG contribute to the integration of working memory processes necessary for maintaining and retrieving the beat, especially in more challenging rhythmic contexts.

Building on earlier findings highlighting the basal ganglia’s involvement in beat strength modulation, Koshimori et al. [70] further investigated this region by comparing dopamine responses during auditory-paced and unpaced finger tapping using PET imaging. They found that rhythmic auditory stimulation (RAS) significantly improved task performance, reducing both the absolute tapping period error and its variability. Interestingly, when auditory cues were absent, there was a higher dopamine response in the left ventral striatum, which may be attributed to the increased motivational and attentional demands of the task. Additionally, the study found that RAS reduced the variability in binding potential within the basal ganglia. This suggests that RAS may reduce reliance on dopaminergic systems in the basal ganglia during motor tasks, possibly by providing a regular auditory cue that helps the brain regulate its dopamine response more effectively. These findings have direct clinical implications for treating neuromotor diseases, such as Parkinson’s disease. The observed modulation of the dopamine system in the basal ganglia during RAS, including reduced dopamine receptor variability and demand, provides a deeper understanding of how RAS enhances motor function.

Regarding the role of the cerebellum, Kung et al. [64] found that the activity in lobule VIII was negatively correlated with beat tapping performance, with greater activity being observed for metrically complex rhythms. This finding corroborates the notion that the cerebellum is involved in the production of complex motor responses, error correction, and feedback processing [71,72,73]. Moreover, Baer et al. [74] found an association between regional cerebellar volumes, finger-tapping performance, and early musical training, particularly in those who began training before age 7. Specifically, participants with early musical training had reduced white matter volume in both cerebellar hemispheres and in specific cerebellar regions (right lobules IV, V, and VI) compared to those who started musical training later. Enhanced timing abilities were specifically associated with reduced volumes in the right lobule VI, suggesting that smaller cerebellar volumes observed in musicians may reflect more precise motor control and coordination in rhythmic tasks.

While MRI and PET studies have provided valuable information about the brain’s structural and functional responses during sensorimotor synchronization, additional evidence of the neural networks involved has been obtained through functional near-infrared spectroscopy (fNIRS) studies. This non-invasive neuroimaging tool measures changes in hemoglobin (Hb) concentrations within the brain in freely moving participants. Unlike MRI, this technique offers two significant ecological advantages: it allows for unrestricted movement and eliminates the noise artifacts associated with MIR scanners, making it particularly well suited for sensorimotor studies.

In a study by Koenraadt et al. [75], changes in oxy-hemoglobin (HbO) and deoxyhemoglobin (HbR) concentrations were measured while participants tapped at single rates and mixed frequencies. The results showed that tapping at mixed frequencies led to increased motor cortex activity, indicated by a larger HbO increase and a significantly larger HbR decrease compared to low- and high-frequency tasks. The study suggests that this increased activity during less predictable tapping is due to the recruitment of a voluntary command motor system, contrasting with the more automated control used in single-frequency tapping. In another study, the researchers investigated the effects of 1 h rhythmic auditory cue training on both behavioral performance and hemodynamic responses measured using fNIRS [76]. The participants engaged in bilateral tapping to rhythmic auditory cues using an electronic drum (e-drum). The results demonstrated that the training improved beat regularity and decreased the variability in tapping intervals across participants. Hemodynamically, there was a significant reduction in HbO concentration and a corresponding decrease in HbR concentration, primarily in the premotor areas. Notably, the study found that the participants who showed the most improvement in behavioral performance exhibited a smaller reduction in HbO concentration, suggesting that these individuals maintained higher levels of cortical activity to support enhanced motor performance.

Together, these studies demonstrate that auditory–motor entrainment engages bilateral cortical and subcortical networks and alters biochemical response systems. This highlights the intricate interplay between cognitive processes such as beat perception, working memory, and motor planning, as well as their integration with motor execution in regions, namely the ventrolateral prefrontal cortex, basal ganglia, and cerebellum. Additionally, they suggest that hemodynamic activity in cortical motor and premotor areas is modulated by task complexity and the level of practice, emphasizing the plasticity of these neural circuits in response to training and experience.

### 3.3. Studies Employing Perturbation Approaches and Combined Neuroimaging Techniques

In recent years, neurostimulation techniques, such as transcranial magnetic stimulation (TMS) and transcranial direct current stimulation (tDCS), have emerged as relevant tools in scientific inquiry, enabling the temporary modulation of neural activity to observe its effects on behavior. Building on evidence from neuroimaging studies, there is a growing interest in using neurostimulation to explore the complex mechanisms underlying sensorimotor synchronization by probing the causal relationships between specific brain regions and sensorimotor abilities.

Giovannelli and colleagues [77] delivered low-frequency (1 Hz) repetitive transcranial magnetic stimulation (rTMS) to the right dPMC, the SMA, and a control midline occipital site on four separate days. During each session, participants performed a synchronization–continuation task that involved isochronous, metrical, and non-metrical sequences. The study found that synchronization accuracy was significantly affected only when the right dPMC was transiently perturbed by the rTMS, while no changes observed upon stimulation of the SMA, left dPMC, or the midline occipital area. The disruption was more pronounced in complex metrical rhythms and was absent in non-metrical and isochronous sequences. Importantly, the disruption was specific to the auditory–motor synchronization process, as rTMS to the right dPMC selectively impaired the synchronization of taps to external cues without affecting the inter-tap interval (ITI), indicating that the right dPMC plays a crucial role in mediating AMS.

Pollok et al. [78] examined the role of the left dorsolateral premotor cortex (dPMC) by applying transcranial direct current stimulation (tDCS) for 10 min. Participants received either anodal, cathodal, or sham stimulation while performing a synchronization–continuation task and a simple reaction time task. The study demonstrated that modulating the excitability of the left dPMC specifically affected the timing of movements during the continuation task, with cathodal tDCS significantly decreasing the inter-tap intervals and anodal tDCS showing a trend toward increasing ITIs. Since neither the reaction time nor the synchronization phase of the tapping task was affected by tDCS, these findings suggest that different cortical areas within the motor control network contribute distinctively to different aspects of movement timing, particularly in the context of rhythm reproduction. This result thus highlights the role of the left dPMC in precise rhythm reproduction.

Finally, two studies employed fMRI in combination with either EEG [79] or MEG [80] to investigate how SMA activity is modulated by movement rate and internal timing mechanisms. Both studies used a finger tapping task that involved either continuous synchronization (fast tapping) or tapping on every fourth beat (slow tapping). Gompf et al. [79] found a complex, task-dependent relationship between brain activity (measured by the BOLD signal in fMRI) and beta oscillations in the SMA. During both fast and slow tapping tasks, the BOLD signal increased (indicating more activity in the SMA) while beta power decreased (indicating desynchronization). However, in tasks that required more internal timing (slow tapping), there was a smaller decrease in low beta oscillations, suggesting that beta rhythms might play different roles depending on the task demands. This study highlights the complex and task-dependent role of beta rhythms in the SMA, particularly in modulating internal timing demands during movement.

Pflug et al. [80] reported that interactions between the auditory and motor cortices during tapping were strongest in the low beta band, emphasizing the role of beta oscillations in coordinating sensory and motor functions. The study also found that the left auditory cortex preferentially tracked faster rhythms while the right auditory cortex was more involved in processing slower, internally generated rhythms. These findings highlight the significant contribution of sensory cortices in the hemispheric specialization of motor responses, with beta oscillations being crucial for the integration of sensory input with motor actions [79,80].

Together, these studies highlight the distinct and critical roles that specific brain regions, such as the dPMC and SMA, play in sensorimotor synchronization. Through neurostimulation techniques like TMS and tDCS, researchers have demonstrated that the right dPMC is crucial for auditory–motor synchronization, particularly in complex rhythmic tasks, while the left dPMC is more involved in the precise reproduction of rhythmic sequences. Additionally, the integration of fMRI with EEG and MEG has revealed a complex relationship between BOLD activity and beta oscillations in the SMA, with beta rhythms playing different roles in coordinating sensory and motor functions and demonstrating clear hemispheric specializations in processing different rhythmic patterns.

**Table 2:** Key characteristics and findings of the included studies.

Author(s)	Sample (*n*) Female/Male	Age (M or Range)	Method	Task(s)	Key Findings
*Electroencephalography (EEG) studies*					
Bavassi et al., 2017 [55]	13 1F/12M	20–31	EEG	AMS	The correction of a large asynchrony + the recovery after a perturbation → similar neural mechanisms
Crasta et al., 2018 [49]	40 23F/18M	23.78 (18–39)	EEG	AMS Self-paced Listening	Listening before AMS: ↓ evoked power associated with sensorimotor processing (4–20 Hz) and anticipation and predictive timing (13–16 Hz)
D’Andrea-Penna et al., 2020 [56]	35 20F/15M	19 (18–24)	EEG	AMS (oddball paradigm) Listening	Deviant beats: ↑ N1-P2-evoked potentials which were positively correlated with synchronization accuracy
De Pretto et al., 2018 [51]	14 7F/7M	27.7	EEG	AMS Self-paced Listening	AMS vs. self-paced: ↑ left IFG Self-paced vs. AMS: ↑ bilateral IPL IFG → beat-based auditory–motor coupling
Mathias et al., 2020 [57]	29 21F/8M	22.6 (18–30)	EEG	AMS (1:1, 1:2, 3:2) Motor condition Listening	AMS and listening: ↑ complexity ↓ entrainment AMS vs. listening: ↑ EEG spectral density
Nijhuis et al., 2021 [53]	20 7F/13M	28.35	EEG	AMS, VMS AMS, VMS (imagined taps) Listening	Imagined and AMS (visual): cortico-muscular connectivity time-locked to synchronized taps Imagined vs. AMS taps: ↓ amplitude
Nozaradan et al., 2015 [50]	8 3F/5M	27 (22–36)	EEG	AMS (RH) AMS (LH) Listening	2.4 Hz SS-EP → beat-related entrainment 1.2 Hz SS-EP → movement-related entrainment 3.6 Hz SS-EP → nonlinear sensorimotor integration
Rosso et al., 2021 [54]	28 18F/10M	29.07	EEG	AMS	↑ neural stability = ↑ synchronization accuracy → left centroparietal cluster + left temporal electrodes
Stegemöller et al., 2018 [58]	32 15F/17M	23	EEG	AMS (tone-only) AMS (music-slow) AMS (music-fast)	AMS with music vs. tone-only → SM1 and SMA Fast AMS ↑ alpha power Slow AMS ↑ beta band power
Stegemöller et al., 2021 [59]	20 11F/9M	23	EEG	AMS (tone-only) Relaxing music-slow Activating music-fast	Relaxing music vs. tone-only ↑ power Activating vs. relaxing music ↓ alpha and beta power
*Magnetic resonance imaging (MRI), Functional near-infrared spectroscopy (fNIRS), and Positron emission tomography (PET) studies*					
Baer et al., 2015 [74]	31, 13F M = 20, 6F/14M NonMus = 11, 7F/4M	Mus = 26.3 NonMus = 25.6	fMRI	AMS Unpaced tapping (SCP)	↓ cerebellar volume associated with ↑ timing skills, ↑ musical experience, and earlier music education ↑ timing performance ↓ volume in right lobule VI
Blecher et al., 2016 [69]	20 11F/9M	29.47 (19–40)	dMRI	AMS AMS (changes in metric structure)	↑ FA in the left arcuate fasciculus and temporal callosal fibers positively correlated with ↑ negative asynchrony = ↑ synchronization abilities
Curzel et al., 2021 [76]	26 19F/7M	21.27	fNIRS	AMS (bilateral tapping) Listening	AMS: ↓ activity in premotor areas ↑ behavioral performance correlated with the smallest ↓ in brain activity
Koenraadt et al., 2013 [75]	11 9F/2M	30 (18–54)	fNIRS	AMS (low, mid, and high frequency) AMS at mixed frequencies	Mixed vs. single-frequency task: ↑ HbO and ↓ HbR in the primary and premotor cortices Mid-frequency: smaller HbR ↓ than the mixed task
Koshimori et al., 2019 [70]	8 4F/4M	27.25	PET	AMS Unpaced tapping	AMS: ↓ binding potential variability Unpaced: ↑ dopamine response in the left VS
Kung et al., 2013 [64]	11 5F/6M	24.7 (20–38)	fMRI	AMS AMS (finding the beat)	Both conditions: STG, premotor cortex, BG, VLPFC AMS: STG and VLPFC → positively correlated; Cerebellum (lobule VIII) → negatively correlated
Miyata et al., 2022 [68]	18 10F/8M	21.4 (19–27)	fMRI	AMS AMS (tempo change) AMS (random)	Temporal prediction: the SMA, right IFG, STG, and left cerebellum; bilateral dPMCexplains the individual variation in prediction ability
Witt and Stevens, 2013 [66]	45 28F/17M	20.3 (12–43)	fMRI	AMS Unpaced tapping (SCP) Listening	Unpaced tapping: ↑ activity in the right VLPFC ↑ consistency = top-down control (motor and sensory) ↓ consistency = bottom-up control (sensory-driven)
*Perturbation approaches and combined neuroimaging techniques*					
Giovannelli et al., 2014 [77]	14 9F/5M	23.9 (21–30)	TMS	AMS (isochronous, patterned) SCP	AMS accuracy mediated by the right dPMC AMS → SMA, left dPMC, midline occipital area
Gompf et al., 2017 [79]	25 15F/10M	23.8 (19–31)	fMRI + EEG	AMS—Fast (every beat) AMS—Slow (every 4th beat)	Both tasks: ↑ BOLD activity in the SMA ↓ in the beta band = task-independent nonlinear relationship
Pflug et al., 2019 [80]	fMRI = 25, 15F/10M MEG = 17, 11F/6M	fMRI = 24 (19–31) MEG = 26 (21–38)	fMRI + MEG	AMS—Fast (every beat) AMS—Slow (tap on every fourth beat)	Faster rhythm → the left AC Slower rhythm → the right AC Motor cortices and the cerebellum → the temporal regularities of the motor output
Pollok et al., 2017 [78]	18 9F/9M	22.8 (21–27)	tDCS	AMS SCP Reaction time task	Unpaced: cathodal tDCS ↓ ITIs, anodal tDCS ↑ ITIs; Left dPMC → involved in rhythm reproduction (internal timing), but not in synchronization

*Notes:* AMS (auditory–motor synchronization) denotes isochronous tapping tasks to auditory stimuli. AC = auditory cortex; BG = basal ganglia; BOLD = blood-oxygen-level-dependent; dMRI = diffusion magnetic resonance imaging; dPMC = dorsal premotor cortex; EEG = electroencephalography; F = female; FA = fractional anisotropy; fMRI = functional magnetic resonance imaging; fNIRS = functional near-infrared spectroscopy; HbO = oxy-hemoglobin; HbR = deoxy-hemoglobin; IFG = inferior frontal gyrus; IPL = inferior parietal lobule; ITI = inter-tap interval; LH = left hand; M = male; MEG = magnetoencephalography; Mus = musician; NonMus = non-musician; PET = positron emission tomography; RH = right hand; SCP = synchronization continuation paradigm SM1 = primary sensorimotor cortex; SMA = supplementary motor area; SS-EP = steady-state-evoked potentials; STG = superior temporal gyrus; tDCS = transcranial direct current stimulation; TMS = transcranial magnetic stimulation; VLPFC = ventrolateral prefrontal cortex; VMS = visuo-motor synchronization; VS = ventral striatum.

## 4. Discussion

**Figure 2:**
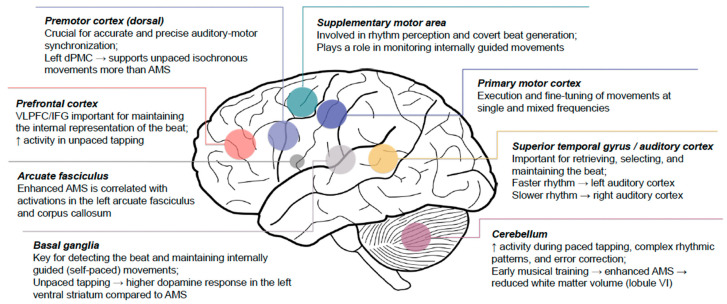
Key brain regions involved in auditory–motor synchronization (AMS).

Temporally synchronizing motor actions with predictable external events is fundamental across various activities—from aligning simple finger movements with metronome clicks to dancing to music and playing in a musical ensemble. Recent advances in neuroscience have enabled researchers to delve deeper into understanding the intricate neural mechanisms underlying sensorimotor synchronization by employing neuroimaging techniques with varying temporal and spatial properties. This scoping review identified 22 studies published in the last decade, extending the earlier reviews on auditory–motor research using finger-tapping paradigms [3,30]. These studies reflect advances in the field and demonstrate the complex interplay between various cortico–subcortical structures involved in auditory–motor entrainment and synchronization (see Figure 2). The following sections discuss the key neural mechanisms involved in AMS and the clinical implications of rhythmic entrainment.

### 4.1. Temporal and Spatial Characteristics of Auditory–Motor Synchronization

Earlier EEG studies have shown that the oscillatory neural patterns within the beta and gamma frequency bands are closely tied to the anticipation and execution of movements [81,82] and are, therefore, of significant interest to researchers investigating the relationship between the auditory and motor systems. Specifically, it has been proposed that beta amplitude decreases prior to and during movement execution, followed by a rebound after task completion [83,84], while increased gamma band activity has been observed in anticipation of auditory stimulus onset, with evoked gamma-band responses occurring in response to the stimuli [85]. Beta oscillations play a critical role not only in movement execution but also in maintaining synchronization accuracy. For instance, stability in beta oscillatory activity has been linked to more precise motor timing [54]. Recent research also suggests that the relationship between beta oscillations and BOLD activity is task-dependent, wherein varying rhythmic complexities modulate the neural oscillations in distinct ways [79,80].

Notably, studies have shown that even passive listening to isochronous sound stimuli modulates both power and phase coherence in beta-band oscillations across several cortico–subcortical areas, including the sensorimotor cortex, inferior frontal gyrus, supplementary motor area, and the cerebellum [41]. These modulations reflect the brain’s preparatory and predictive processes in response to rhythmic auditory cues. The studies reviewed here further indicate that listening to auditory rhythms before producing rhythmic movements improves auditory–motor entrainment [49]. This enhancement suggests a priming effect, where exposure to rhythm improves the brain’s ability to synchronize motor actions with auditory stimuli, providing an additional rationale for incorporating rhythm-based interventions in clinical settings.

Over the past three decades, a substantial body of literature has focused on identifying the spatial characteristics of different timing processes, such as beat extraction, stimuli periodic structure, timing and adjusting one’s movements in relation to an external rhythmic cue, as well as retaining the temporal information of an auditory beat that is no longer available. Collectively, our findings are in line with earlier reviews of MRI studies which demonstrated that both synchronization and continuation tapping involve a large, distributed network of cortical and subcortical areas such as the sensorimotor cortex, premotor cortex, SMA, basal ganglia, and cerebellum [60,63]. This network is bilaterally distributed, with significant contributions from both hemispheres in processing rhythms and ensuring precise timing during auditory–motor synchronization [61].

Specifically, the SMA is involved in covert beat generation, essential for tasks such as self-paced and continuation tapping, where an internal representation of the beat needs to be maintained in the absence of external cues [86]. In addition, the pre-SMA is thought to play a role in preparing and monitoring internally guided movements and self-paced tapping [60,63,67,87,88]. The SMA has also been recognized as an important cortical node in the basal ganglia-thalamus-cortical loops in motor control and beat-based timing [72,86]. This network supports the integration of sensory input and motor planning, ensuring the precise timing needed for effective auditory–motor synchronization.

The included studies suggest that the basal ganglia play a pivotal role in beat-based timing [64,88]. For instance, the basal ganglia, particularly the putamen and globus pallidus, are key for detecting and predicting the beat as well as for internally guided rhythmic movements. Furthermore, the basal ganglia and cerebellum seem to be involved in distinct but complementary aspects of timing and sensorimotor synchronization [61,72,89], with the cerebellum playing a more significant role in externally guided or paced movements and the basal ganglia being more involved in processing rhythms with a clear beat structure [29,60,61,64]. For example, Kung et al. [64] observed an increase in cerebellum activity (lobule VIII) during tapping to the beat of complex rhythmic sequences compared to isochronous tones [29].

Furthermore, recent advancements in neuroimaging and neurostimulation techniques have enabled researchers to directly probe the causal roles of specific brain regions in AMS. Unlike previous studies that relied primarily on correlational data, these new approaches allow for a more detailed understanding of how cortical and subcortical regions interact to support the timing and synchronization processes essential for AMS [90]. Studies using TMS and tDCS have demonstrated that the right dPMC is crucial for auditory–motor synchronization, particularly in complex rhythmic tasks, while the left dPMC is more involved in the precise reproduction of rhythmic sequences [77,78]. Hemispheric specialization in rhythmic processing has also been observed in the auditory cortex, with the left auditory cortex preferentially processing faster rhythms while the right cortex is more involved in slower, internally generated rhythms [80]. This highlights the importance of considering hemispheric specialization when examining the neural mechanisms underlying AMS.

Taken together, recent research has significantly advanced our understanding of the neural mechanisms underlying auditory–motor entrainment and synchronization. The integration of neuroimaging and neurostimulation techniques has provided causal evidence for the roles of specific brain regions in AMS, particularly the SMA and dPMC [77,78]. Additionally, the exploration of hemispheric specialization in rhythmic processing has revealed the distinct contributions of the left and right hemispheres, adding a new dimension to our understanding of AMS [80], while more recent studies have clarified the complementary roles of the cerebellum and basal ganglia in processing different rhythmic structures [64]. Finally, recent findings indicate that the cerebellum plays an important role in tracking and processing rhythmic patterns with a less predictable beat structure, which contrasts with the basal ganglia’s role, particularly the putamen, in processing rhythms with a clear beat structure. This hypothesis was further corroborated by a recent meta-analysis [61].

### 4.2. Clinical Implications of Rhythmic Auditory–Motor Entrainment

Understanding the mechanisms underlying auditory–motor entrainment is of particular relevance for designing targeted music-based interventions in movement disorders, as well as neurodevelopmental conditions. Indeed, impairments in rhythm processing and production, including poor performance on AMS tasks, have been consistently reported in motor disorders [27,91,92,93,94], attention disorders [95], and developmental speech and language disorders (see [96,97] for reviews). For instance, individuals with Parkinson’s disease experience significant deficits across different rhythmic movements, as observed in increased manual, orofacial, and gait variability [94,98,99,100]. Heightened tapping variability is also observed throughout the manifest and premanifest stages of Huntington’s disease, with poorer performance on continuation tapping being significantly associated with disease progression [101,102,103,104].

Growing research has reported that children with language and movement disorders often present difficulties in rhythm perception and production tasks, as shown by increased variability in paced tapping tasks to a metronome or musical excerpts [105,106,107,108]. Moreover, Lense et al. [109] noted that impairments in rhythmic synchronization to beat-based stimuli are observed across various neurodevelopmental disorders (e.g., attention-deficit/hyperactivity disorder, dyslexia, autism spectrum disorder), suggesting that rhythmic timing vulnerabilities may be a transdiagnostic marker. Collectively, these findings indicate that common mechanisms underlying temporal hierarchical processing and prediction are crucial for extracting temporal regularities in stimuli such as music and language, as well as for the production of timed movements [96,110].

There is ample evidence that the presentation of external rhythmic auditory stimuli significantly improves movement control in clinical populations with motor impairments, such as Parkinson’s disease [111,112], traumatic brain injury [113,114], stroke [115], developmental coordination disorder [106], and cerebral palsy [116]. Rhythmic auditory cues induce auditory–motor entrainment by providing continuous external temporal references, generating expectations for when the next auditory event will occur. These predictions enable movement planning and anticipation, priming the motor system and consequently improving the quality and precision of the motor responses [7]. It has been consistently demonstrated that rhythm-based rehabilitation techniques, such as rhythmic auditory stimulation^®^, promote long-term improvement in gait spatiotemporal parameters, enhancing gait velocity and stride length as well as reducing gait cadence (for a review, see [116,117,118,119]). Research also indicates that rhythm-based training enhances performance in sensorimotor synchronization and rhythm perception tasks for individuals with Parkinson’s disease [112], with additional studies demonstrating improvements in upper limb function post-stroke [119].

Studies have also demonstrated the benefits of rhythmic priming and rhythmic cueing across language and speech disorders [120]. Regular rhythmic stimuli presented before a set of naturally spoken sentences improved sentence repetition in children with developmental language disorder and with typical development [121,122]. Additionally, rhythmic cueing has been shown to benefit speech fluency for individuals who stutter by regulating the temporal structure of the words [123,124,125]. Furthermore, grammatical processing is significantly improved for children with dyslexia, developmental language disorder, and specific language impairment when a regular rhythmic primer is presented compared to irregular rhythms or other sounds [126,127]. The implications of these findings are further supported by recent studies indicating that patients might benefit from exercises that gradually increase rhythmic complexity [57,64], which may help re-engage or strengthen the underlying neural circuits [61]. These clinical applications highlight the potential of rhythm-based interventions to improve not only motor control but also cognitive and linguistic functions, further broadening the scope of auditory–motor entrainment as a therapeutic tool.

The findings presented here also suggest that rehabilitation strategies could benefit from considering hemispheric specialization, leveraging the distinct contributions of each hemisphere to enhance therapeutic outcomes in motor and cognitive rehabilitation [77,78,80]. Together, the effectiveness of rhythm-based interventions demonstrates that external rhythmic stimuli can improve attention allocation and temporal predictions and promote sensorimotor coupling by entraining auditory–motor networks [7,96].

### 4.3. Limitations and Future Directions

Although this review focused specifically on finger tapping studies, we encountered heterogeneity in task designs and conditions across the included studies. This variability posed some challenges to identifying distinct neural networks involved in paced versus unpaced tapping. Future studies should aim to carefully document task-specific variables to allow for better comparisons across studies. While this was outside the scope of the current review, future research should examine more complex synchronization patterns involving the locomotion system, such as walking or dancing. Locomotion remains largely underexplored in the context of AMS (see [4]) despite being the most evolutionarily conserved rhythmic activity in humans and other species. Investigating how these more naturalistic and dynamic forms of movement engage AMS networks could reveal new aspects of the neural mechanisms supporting synchronization. Such research could bridge the gap between fine motor tasks and whole-body rhythmic activities, providing a more comprehensive understanding of AMS in real-world contexts.

As suggested by Kasdan et al. [61], future studies should consider using various baseline conditions that control for basic auditory, motor, attentional, or other cognitive processes (e.g., non-beat-based rhythms) rather than relying on a baseline of rest or silence. Choosing appropriate baseline conditions will help delineate more specific brain networks involved in musical entrainment. Incorporating baselines that account for rhythmic complexity, as discussed in studies like Mathias et al. [57] and Kung et al. [64], could provide more nuanced insights into how different brain regions contribute to AMS. While most of the existing literature has employed EEG and MRI, there is growing interest in using combined methods that are non-invasive, portable, and suited for collecting data in ecological settings, such as integrated EEG-fNIRS systems. The use of such multimodal approaches can further enhance our understanding of the temporospatial characteristics of AMS across diverse clinical populations and settings.

Lastly, while our findings contribute to the growing understanding of the neural mechanisms underlying auditory–motor entrainment in neurotypical adults, the developmental trajectories of auditory–motor synchronization in atypical neurodevelopment remain less explored. Gaining insight into how these neurobiological mechanisms evolve across a lifespan could inform the development of individualized rhythm-based treatments in clinical settings and inform tailored neurorehabilitation strategies.

## 5. Conclusions

Over the past few decades, researchers have made significant progress in elucidating the link between behavioral and neurobiological mechanisms underlying auditory–motor entrainment and synchronization. The current review synthesizes findings from neuroimaging and neurostimulation methods, emphasizing the multifaceted nature of AMS. The evidence reviewed here is particularly relevant for interventions in sensorimotor domains, such as gait and upper limb rehabilitation in Parkinson’s disease and stroke, as well as neurodevelopmental conditions. Accurately mapping the underlying neural networks that mediate auditory–motor entrainment could provide valuable biomarkers for diagnostic purposes and help guide the development of individually tailored neurorehabilitation strategies.

## Figures and Tables

**Table 1:** An example table of searches entered into the MEDLINE database.

Database	MEDLINE (Ovid)		
**Coverage**	Medicine and Health		
**Limits**	Publication Type: “Article”, Language: “English”		
	***Population*** **Neurotypical adults**	***Concept*** **Entrainment/AMS**	***Context*** **Finger-tapping paradigm**
	((healthy or neurotypical) adj3 (individual* or subject* or participant* or adult*)).mp.	((“rhythmic auditory” or “auditory motor” or “rhythmic synchronization” or auditory) adj3 (cue* or stimulation or entrain* or coupling or integrat* or synchron*)).mp.	(“finger tapping” or “hand tapping” or “rhythmic movement” or “motor performance” or “motor control”).mp.
**Results**	294		

*Note*: In the MEDLINE database, the asterisk (*) serves as a truncation tool, enabling the retrieval of all terms that share a common root.

## Data Availability

Not applicable.

## Supplementary Materials

The following supporting information can be downloaded at: https://www.mdpi.com/article/10.3390/brainsci14111063/s1, Table S1: Search Strategy.
