# Supplementary material for: From Sound to Movement: Mapping the Neural Mechanisms of Auditory–Motor Entrainment and Synchronization
Source: Brain Sci. 2024 Oct 25;14(11):1063. doi: 10.3390/brainsci14111063 (PMC11592450; doi:10.3390/brainsci14111063)
Supplement: Supplementary file 1 [file brainsci-14-01063-s001.zip › brainsci-3260284-supplementary.pdf]

Supplementary Table S1. Search Strategy.

**Database**	**Medline** (Ovid)		
**Coverage**	Medicine and Health		
**Limits**	Publication Type: “Article”, Language: “English”		
**Search Query**	**Population: Neurotypical adults**	**Concept: Entrainment/AMS**	**Context: Finger-tapping paradigm**
	Keywords	*Keywords*	*Keywords*
	((healthy or neurotypical) adj3 (individual* or subject* or participant* or adult*)).mp.	(("rhythmic auditory" or "auditory motor" or "rhythmic synchronization" or "rhythmic entrainment" or auditory) adj3 (cue* or stimulation or entrain* or coupling or integrat* or synchron*)).mp.	("finger tapping” or "hand tapping" or “rhythmic movement” or “motor performance" or “motor control").mp.
**Results**	294		

**Database**	**Embase** (Ovid)		
**Coverage**	Medicine and Health		
**Limits**	Publication Type: “Article”, Language: “English”		
**Search Query**	**Population: Neurotypical adults**	**Concept: Entrainment/AMS**	**Context: Finger-tapping paradigm**
	Keywords	*Keywords*	*Keywords*
	((healthy or neurotypical) adj3 (individual* or subject* or participant* or children or adult*)).mp.	"rhythmic auditory" or "auditory motor" or "rhythmic synchronization" or "rhythmic entrainment" or auditory) adj3 (cue* or stimulation or entrain* or coupling or integrat* or synchron*)).mp.	"motor control" or "motor learning" or motor or "hand tapping" or "finger tapping” OR “motor performance").mp.
**Results**	343		

**Database**	**PsycInfo** (Ovid)		
**Coverage**	Psychology		
**Limits**	Publication Type: “Article”, Language: “English”		
**Search Query**	**Population: Neurotypical adults**	**Concept: Entrainment/AMS**	**Context: Finger-tapping paradigm**
	Keywords	*Keywords*	*Keywords*

	((healthy or neurotypical) adj3 (individual* or subject* or participant* or children or adult*)).mp.	"rhythmic auditory" or "auditory motor" or "rhythmic synchronization" or "rhythmic entrainment" or auditory) adj3 (cue* or stimulation or entrain* or coupling or integrat* or synchron*)).mp	"motor control" or "motor learning" or motor or "hand tapping" or "finger tapping" OR "motor performance").mp.
**Results**	326		

**Database**	**Scopus**		
**Coverage**	Interdisciplinary		
**Limits**	Publication Type: "Article", Language: "English"		
**Search Query**	**Population: Neurotypical adults**	**Concept: Entrainment/AMS**	**Context: Finger-tapping paradigm**
	Keywords	*Keywords*	*Keywords*
	"healthy participants" OR "neurotypical" OR "healthy subject" OR "healthy children" OR healthy	"rhythmic auditory" OR "auditory motor" OR "rhythmic synchronization" OR "rhythmic entrainment")) OR (TITLE-ABS KEY ("auditory stimulation" OR "acoustic stimulation"	"motor control" OR "motor learning" OR "hand tapping" OR "finger tapping" OR "motor performance"
**Results**	467		
